# Insights into hydro thermal gasification process of microplastic polyethylene via reactive molecular dynamics simulations

**DOI:** 10.1038/s41598-024-69337-z

**Published:** 2024-08-13

**Authors:** Do Tuong Ha, Hien Duy Tong, Thuat T. Trinh

**Affiliations:** 1 Faculty of Applied Sciences, Ton Duc Thang University, Ho Chi Minh City, Vietnam; 2 Faculty of Engineering, Vietnamese-German University (VGU), Thu Dau Mot City, Binh Duong Province Vietnam; 3 Porelab, Department of Chemistry, Norwegian University of Science and Technology, NTNU, Trondheim, 7491 Norway

**Keywords:** Pollution remediation, Chemical engineering, Energy, Environmental chemistry, Theoretical chemistry

## Abstract

Microplastics have become a pressing environmental issue due to their widespread presence in our ecosystems. Among various plastic components, polyethylene (PE) is a prevalent and persistent contaminant. Hydrothermal gasification (HTG), a promising technology for converting PE into syngas, holds great promise for mitigating the microplastic problem. In this study, we employ ReaxFF molecular dynamics simulations to investigate the HTG process of PE, shedding light on the intricate relationships between temperature, water content, carbon conversion efficiency, and product distributions. The results reveal that hydrothermal gasification of PE is a complex process involving multiple reaction pathways. Consistently with experimental findings, the calculations indicate that the gas phase exhibits a substantial hydrogen fraction, reaching up to 70%. Interestingly, our simulations reveal a dual role of water content in the HTG process. On one hand, water enhances hydrogen production by promoting the gas formation. On the other hand, it elevates the activation energy required for PE decomposition. Depending on the water content, the calculated activation energies range from 176 to 268 kJ/mol, which are significantly lower than those reported for thermal gasification (TG). This suggests that HTG may be a more efficient route for PE conversion. Furthermore, this study highlights the importance of optimizing both temperature and water content in HTG systems to achieve high yields of hydrogen-rich syngas. The results obtained from our ReaxFF MD simulations demonstrate the robustness of this computational methodology in elucidating complex chemical reactions under extreme conditions. Our findings offer critical insights into the design of advanced waste management strategies for microplastics and contribute to the development of sustainable practices for resource recovery. This work underscores the potential of HTG as a key technology for addressing the global challenge of plastic pollution.

## Introduction

Plastic waste management has emerged as a critical global issue due to its significant environmental, health, and economic impacts^1,2^. The negative impacts of microplastic pollution are far-reaching and include disruption of aquatic food chains, harm to marine life, and contamination of drinking water sources. Moreover, exposure to microplastics has been linked to adverse health effects in humans, including respiratory issues, gastrointestinal problems, and reproductive complications. Addressing this growing crisis requires a multi-faceted approach that includes reducing the production of single-use plastics, improving waste management practices, and finding innovative solutions for microplastic removal from the environment.

Addressing the issue of plastic waste requires various solutions, such as material reduction, recyclability design, increased recycling capacity, development of bio-based feedstocks, littering reduction, and green chemistry life-cycle analyses^1,2^. Among these, recycling is particularly significant for reducing plastic waste’s environmental and health impacts^3^. Recycling offers benefits including decreased oil usage, reduced carbon emissions, less waste disposal, and circular economy contribution via resource reuse^3^. Nevertheless, enhancing recycling technology and systems is necessary for improved efficiency, cost-effectiveness, and better handling of diverse plastic waste forms^4^.

Several effective strategies for managing plastic waste such as catalytic degradation and pyrolysis into fuel and developing eco-friendly bio-degradable/bio-based polymers was proposed^5,6^. Catalytic degradation involves breaking down polymer chains into smaller molecules^7–9^. For example, ruthenium supported on zirconia has shown promising results for hydrogenolysis of polyethylene, as it suppresses methane formation and produces a product distribution in the diesel and wax/lubricant base-oil range^7^. The pyrolysis process involves the thermal decomposition of plastics in the absence of oxygen, resulting in the formation of liquid hydrocarbon fuels, gases, and solid carbon residues^10,11^. The application of pyrolysis technology offers numerous benefits such as reducing landfill space requirements, lowering greenhouse gas emissions compared to conventional incineration methods, and recovering valuable resources like oil, gas, and carbon-based materials that can be further utilized in various industries^12^.

Another promising solutions for plastic waste are microplastic thermal gasification^13,14^ and hydrothermal gasification^15^. These processes convert microplastics into valuable products such as fuel and fertilizers, while simultaneously reducing its volume. The breakdown of microplastics in thermal gasification occurs in an oxygen-rich environment, generating syngas, leading to a high burning rate due to internal bubbling, multiple micro explosions, and micro jets^13^. This approach can reduce our reliance on fossil fuels and decrease negative impacts associated with climate change. However, the adoption of microplastic gasification as a widespread technology requires overcoming challenges. The development of scalable, cost-effective systems capable of processing significant quantities of microplastics while minimizing emissions remains an open research question^14^. In another process, the hydrothermal gasification of plastics typically occurs under conditions of elevated temperature ($500\,^{\circ }\hbox {C}$ to $750\,^{\circ }\hbox {C}$) and pressure (23–26 MPa). In this process, supercritical water is employed to convert plastics into syngas and smaller species^15^. By exploiting the unique properties of supercritical water, hydrothermal gasification represents an efficient method for plastic waste treatment and valuable resource recovery.

Polyethylene (PE), a versatile, durable, and inexpensive plastic, is prevalent in modern society. However, its long-term environmental persistence presents ecological and public health challenges due to degradation via photo-degradation, hydrolysis, oxidation, and bio-degradation^12^. Microplastics, resulting from weathering and wear, have garnered attention due to their potential impact on human health. These particles can adsorb toxic persistent organic pollutants (POPs), and organochlorine pesticides, acting as vectors for their transport in aquatic ecosystems, causing harm to marine life^16,17^.

The degradation of PE impacts the development of the plastisphere, a distinct microbial ecosystem on plastic debris, inhabited by heterotrophic bacteria, autotrophs, predators, and symbiotic partners^18^. Some members can be opportunistic pathogens or contribute to polymer degradation. However, the plastisphere’s potential as a reservoir for antibiotic-resistant genes and other health risks warrants concern^19^. Additionally, the release of microplastics into the environment poses concerns about their impact on polymer recycling processes. Both mechanical and chemical recycling methods could be impeded by contaminants such as microplastics and persistent organic pollutants (POPs), resulting in lower-quality recycled products or new pollution sources^20^.

Understanding the degradation mechanism of PE is crucial in developing effective strategies to manage microplastic pollution. Molecular dynamics (MD) simulations are an essential tool for gaining insights into this complex process, as they enable researchers to study the behavior of materials and their interactions at the atomic level. Reactive molecular dynamics simulations^21,22^, have become valuable tools for studying reactive chemical systems at the atomic level. The application of ReaxFF has received significant attention due to its potential in various fields^23–30^. For example, Mattsson et al.^31^ used ReaxFF molecular dynamics simulations to investigate the shock behavior of hydrocarbon polymers and found that ReaxFF best agreed with experimental data. Liu et al.^32^ applied ReaxFF MD simulations to study high-density polyethylene pyrolysis, identifying reaction mechanisms and generating pathways for primary gas molecules in reasonable agreement with experimental results. Hong et al.^33^ examined co-pyrolysis behaviors of coal with high-density polyethylene (HDPE) and polystyrene (PS), discovering synergistic effects between coal and plastics, reducing the activation energy for HDPE co-pyrolysis. More recently, Wang et al.^34^ studied biomass and HDPE co-pyrolysis via ReaxFF molecular dynamics simulation and DFT, reporting that hydrocarbon radicals from HDPE interacted with alcoholic groups in cellulose to form long-chain alcohols while suppressing aldehydes and ketones.

While ReaxFF has successfully predicted reaction pathways for many systems, there has been limited research on using this method to understand the hydrothermal gasification process of microplastic polyethylene. As a result, the detailed molecular-level processes that govern the hydrothermal gasification of PE are not well understood, which hinders the development of effective strategies to address microplastic pollution. This work aims to fill this knowledge gap by utilizing ReaxFF simulations to examine the hydrothermal gasification of microplastic PE. By providing insights into the degradation mechanisms, product distribution, and reaction kinetics under diverse temperature and water content, our study will contribute to a more comprehensive understanding of this process. This, in turn, will guide the development of improved microplastic waste management strategies.

## Models and methods

In molecular dynamics (MD) simulations, employing model compounds is a widely adopted strategy to distill the complexity of real-world systems into more manageable representations. These models, while simplified in size and computational demand, are carefully crafted to retain the key structural and dynamical characteristics of the material under investigation. In this work, we adopted a $\hbox {C}_{30}\hbox {H}_{62}$ hydrocarbon chain model to represent microplastic PE. This model offers a balanced approach between accuracy in depicting PE behavior and the computational resources required for extensive simulations. Our study employed a simulation system composed of 50 PE molecules. To explore the effects of water content on the hydrothermal gasification (HTG) process, we conducted a series of simulations where the water content within the simulation box was systematically varied from 8 to 37%. This range of water concentrations allowed us to probe the influence of moisture on the degradation process under extreme conditions. The overall density of our simulated system was kept approximately at 0.2 g/$\hbox {cm}^3$, which is consistent with the gasification process simulations conducted by Pang et al.^35^. The simulation details are summarized in Table 1.

**Table 1:** Model systems for HTG simulation of PE in ReaxFF MD.

System	PE (molecules)	Water (molecules)	No. of atoms	Box length (Å)
1	50 (92%)	100 (8%)	4900	58.11
2	50 (80%)	300 (20%)	5500	60.10
3	50 (70%)	500 (30%)	6100	63.10
4	50 (63%)	700 (37%)	6700	65.10

The number of molecules of PE and water in each system are listed. The value in parentheses (%) show the mass fraction percentage of PE and water.

**Figure 1:**
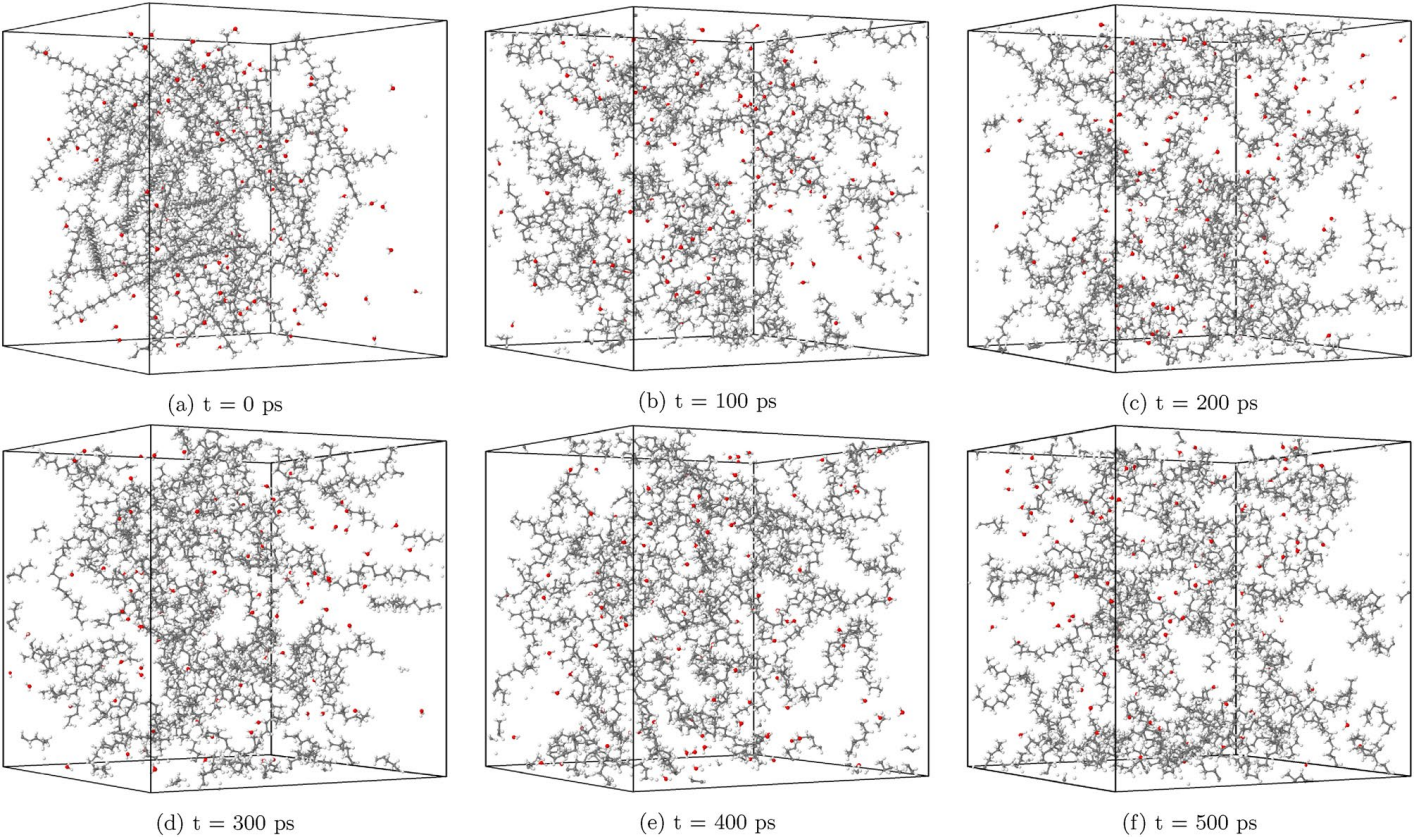
Selected snapshots of the HTG process of PE (system 1) at $\hbox {T} = 1500\,\hbox {K}$ at different simulation times. Carbon, oxygen and hydrogen atoms are depicted in gray, red and white, respectively.

Illustrative snapshots of the simulation system with an 8% water content (system 1) are provided in Fig. 1, offering a visual representation of the configuration used in our ReaxFF MD simulations. These images capture the dynamic interplay between PE molecules and water within the HTG environment, which is critical for understanding the mechanisms governing the gasification process and for informing the design of effective HTG reactors for microplastic waste conversion.

### ReaxFF molecular dynamics and simulation method

The ReaxFF (Reactive Force Field) method was employed for this study due to its ability to accurately describe molecular interactions, particularly chemical reactions. Developed by Adri van Duin and William A. Goddard III, ReaxFF combines molecular mechanics and dynamics with a reactive potential energy function, enabling the modeling of bond breaking and formation. ReaxFF’s ability to adjust bond lengths and angles in response to changing chemical environments makes it a powerful tool for studying molecular-level properties and reactions. For this method, the total energy of a system is expressed in Eq. (1) as follows:

1 $$\begin{aligned} E_{\text {system }}=E_{\text {bond }}+E_{\text {over }}+E_{\text {under }}+E_{\text {val }}+E_{\text {pen }}+ E_{\text {tors }}+E_{\text {conj }}+E_{\text {vdWaals }}+E_{\text {Coulomb }}, \end{aligned}$$

where $E_{\text {system }}$ represents the total energy of the system, and $E_{\text {bond }}$, $E_{\text {over }}$, $E_{\text {under }}$, $E_{\text {val }}$, $E_{\text {pen }}$, $E_{\text {tors }}$, $E_{\text {conj }}$, $E_{\text {vdWaals }}$, and $E_{\text {Coulomb }}$ represent the bond energy based on the bond order, the over-coordination atom energy, the under-coordination atom energy, the valence angle energy, the penalty energy for the double bond, the torsion angle energy, conjugated effect energies, and the van der Waals and Coulomb forces for non-bonding interactions, respectively. For a more detailed description of the ReaxFF force field and its underlying methodology, interested readers are encouraged to refer to the original paper.

In this study, we employed the potential function parameters developed by Vashisth et al.^36^. These parameters have been extensively validated through experimental data and DFT calculations, making them a popular choice for studying reactions in organic molecules. The ReaxFF simulations were performed using LAMMPS^37^, a classical molecular dynamics code with a focus on materials modeling. To ensure the reliability of our ReaxFF MD simulations, we employed well-established procedures for system preparation, equilibration, and production runs^23,25,30,38,39^. Our systems were initially constructed with random configurations, and each system underwent a series of energy minimization and equilibration steps to reach a stable state before the production run. All systems underwent a 5 ps relaxation at 300 K in the NVT ensemble (constant number of particles N, constant volume V, and constant temperature T) to minimize energy before further simulation.

To accelerate reaction kinetics and overcome time and length scale limitations of traditional experimental methods, we simulated the system at higher temperatures than those typically found in experiments. This approach has been previously demonstrated to be effective in thermal chemical process of different organic materials^23,25,30,38,39^. In this study, we employed temperatures ranging from 1500 to 4200 K. The heating rate for our simulations was set at 20 K/ps, which is consistent with the previous ReaxFF study^30^. A time step of 0.25 fs was used, which is small enough to accurately capture system dynamics without significantly increasing computational cost^25,30,38,39^. We utilized an in-house analysis code based on Python language to examine the MD trajectory from the ReaxFF simulations. This code facilitates the identification of elementary reactions and the formation of various products, allowing us to gain comprehensive insights into the complex chemistry associated with the HTG process.

## Results and discussion

### Reaction mechanism

This section presents the complex reaction mechanisms associated with the HTG of PE. This process characterized by a sequence of chemical transformations that lead to a diverse array of gaseous products. The MD simulations also investigate the impact of temperature on the HTG pathways, spanning an extensive thermal range from 1500 to 4200 K.

To visualize the evolution of the system under varying conditions, we present a series of snapshot images capturing the $\hbox {PE}$ decomposition process at different simulation times within a system containing 8% water by weight. Figure 1 illustrates how $\hbox {PE}$ molecules break down into smaller hydrocarbon chains at $\hbox {T} = 1500~\hbox {K}$. At this lower temperature regime, the production of syngas is minimal.

**Figure 2:**
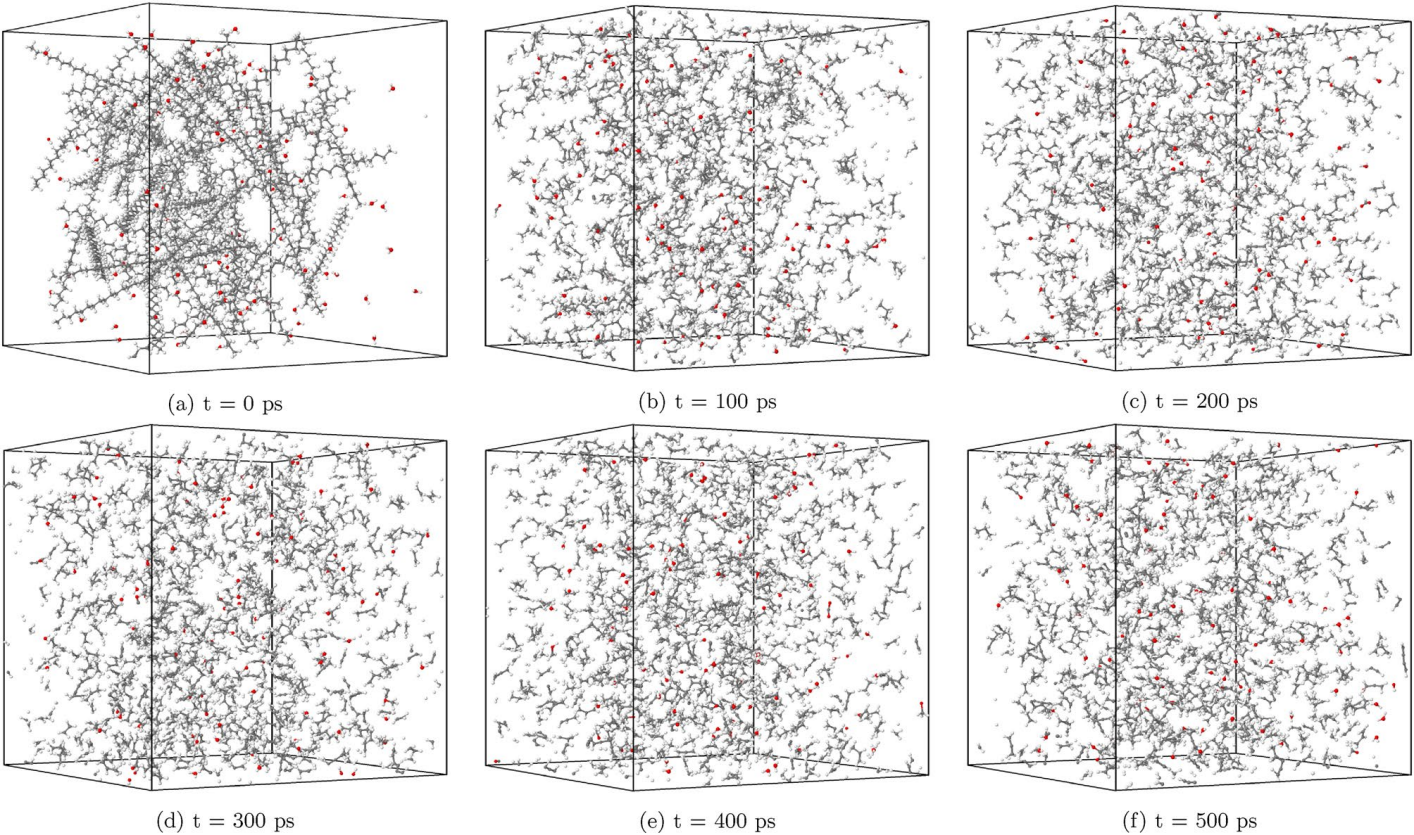
Selected snapshots of the HTG process of PE (system 1) at $\hbox {T} = 2500\,\hbox {K}$ at different simulation times, illustrating the enhanced gas product formation during the HTG process.

Upon increasing the temperature to 2500 K (Fig. 2), we observe an acceleration in the HTG process, accompanied by a significant enhancement in gas product yield. The hydrothermal degradation of $\text {PE}$ at this intermediate temperature begins. Initially, at time $\hbox {t} = 0\,\hbox {ps}$, the $\text {PE}$ is represented as a linear chain of the repeat unit $\hbox {C}_{30}\hbox {H}_{62}$. As the simulation advances, by approximately 100 ps, the chain begins to break down into smaller fragments. The elevated temperatures not only facilitate the generation of hydroxyl radicals but also increase the frequency of chain scission events, leading to the production of a broad spectrum of gases and small hydrocarbon species ranging from C1 to C4. Compared with $\hbox {T} = 1500~\hbox {K}$, the reactions at this temperature results in a substantial increment in syngas yields. The findings highlight the importance of temperature as a critical parameter in controlling product selectivity and yield during the HTG process of $\text {PE}$.

**Table 2:** Representative elementary reactions of PE ($\hbox {C}_{30}\hbox {H}_{62}$) in the HTG process obtained by ReaxFF simulations at $\hbox {T} = 1500~\hbox {K}$.

Nr.	Reactions
1	$\hbox {C}_{30}\hbox {H}_{62} + \hbox {HO}^{\cdot } \rightarrow \hbox {C}_{30}\hbox {H}_{61}^{\cdot } + \hbox {H}_2\hbox {O}$
2	$\hbox {C}_{30}\hbox {H}_{62} + \hbox {H}^{\cdot } \rightarrow \hbox {C}_{22}\hbox {H}_{46} + \hbox {C}_8\hbox {H}_{17}^{\cdot }$
3	$\hbox {C}_{30}\hbox {H}_{62} + \hbox {H}^{\cdot } \rightarrow \hbox {C}_{25}\hbox {H}_{52} + \hbox {C}_5\hbox {H}_{11}^{\cdot }$
4	$\hbox {C}_{30}\hbox {H}_{62} + \hbox {H}^{\cdot } \rightarrow \hbox {C}_{30}\hbox {H}_{61}^{\cdot } + \hbox {H}_2$
5	$\hbox {C}_{30}\hbox {H}_{62} + \hbox {HO}^{\cdot } \rightarrow \hbox {C}_{30}\hbox {H}_{62}\hbox {O} + \hbox {H}^{\cdot }$
6	$\hbox {C}_{30}\hbox {H}_{62} + \hbox {HO}^{\cdot } \rightarrow \hbox {C}_{16}\hbox {H}_{33}^{\cdot } + \hbox {C}_{14}\hbox {H}_{30}\hbox {O}$
7	$\hbox {C}_{30}\hbox {H}_{62} \rightarrow \hbox {C}_{18}\hbox {H}_{37}^{\cdot } + \hbox {C}_{12}\hbox {H}_{25}^{\cdot }$

**Table 3:** Representative elementary reactions of PE ($\hbox {C}_{30}\hbox {H}_{62}$) in the HTG process obtained by ReaxFF simulations at $\hbox {T} = 2500~\hbox {K}$.

Nr.	Reactions
1	$\hbox {C}_{30}\hbox {H}_{62} + \hbox {H}_2\hbox {O} \rightarrow \hbox {C}_2\hbox {H}_{6} + \hbox {C}_{28}\hbox {H}_57^{\cdot } + \hbox {HO}^{\cdot }$
2	$\hbox {C}_{30}\hbox {H}_{62} \rightarrow \hbox {C}_{20}\hbox {H}_{41}^{\cdot } + \hbox {C}_{10}\hbox {H}_{21}^{\cdot }$
3	$\hbox {C}_{30}\hbox {H}_{62} \rightarrow \hbox {C}_{16}\hbox {H}_{33}^{\cdot } + \hbox {C}_{14}\hbox {H}_{29}^{\cdot }$
4	$\hbox {C}_{30}\hbox {H}_{62} + \hbox {H}_2\hbox {O} \rightarrow \hbox {C}_7\hbox {H}_{16} + \hbox {C}_{23}\hbox {H}_{47}\hbox {O}^{\cdot } + \hbox {H}^{\cdot }$
5	$\hbox {C}_{30}\hbox {H}_{62} + \rightarrow$ $\hbox {C}_{11}\hbox {H}_{22} + \hbox {C}_{19}\hbox {H}_{39}^{\cdot } + \hbox {H}^{\cdot }$
6	$\hbox {C}_{30}\hbox {H}_{62} + \hbox {H}^{\cdot } + \hbox {HO}^{\cdot } \rightarrow \hbox {C}_{12}\hbox {H}_{26} + \hbox {C}_{18}\hbox {H}_{36} + \hbox {H}_2\hbox {O}$
7	$\hbox {C}_{30}\hbox {H}_{62} + \hbox {H}^{\cdot } \rightarrow \hbox {C}_{8}\hbox {H}_{18} + \hbox {C}_{22}\hbox {H}_{45}^{\cdot }$
8	$\hbox {C}_{30}\hbox {H}_{62} + \hbox {HO}^{\cdot } \rightarrow \hbox {C}_{30}\hbox {H}_{61}^{\cdot } + \hbox {H}_2\hbox {O}$
9	$\hbox {C}_{30}\hbox {H}_{62} \rightarrow \hbox {C}_{26}\hbox {H}_{53}^{\cdot } + \hbox {C}_4\hbox {H}_9^{\cdot }$
10	$\hbox {C}_{18}\hbox {H}_{37}^{\cdot } + \hbox {C}_{12}\hbox {H}_{25}^{\cdot } \rightarrow \hbox {C}_{30}\hbox {H}_{62}$

The Tables 2 and 3 present a collection of representative elementary reactions obtained through ReaxFF simulations at temperature of 1500 K and 2500 K, respectively. The elementary reactions involved in this process typically consist several steps. Initially, PE interacts with water molecules to form various intermediate products, such as hydrogen radical ($\hbox {H}^{\cdot }$), hydrocarbon ($\hbox {C}_2\hbox {H}_{6}$, $\hbox {C}_7\hbox {H}_{16}$) and free radical species ($\hbox {C}_{10}\hbox {H}_{21}^{\cdot }$, and $\hbox {HO}^{\cdot }$). These radicals then undergo further reactions, leading to the formation of smaller fragments or oligomers (e.g., $\hbox {C}_{28}\hbox {H}_57^{\cdot }$, $\hbox {C}_{16}\hbox {H}_{33}^{\cdot }$, $\hbox {C}_{14}\hbox {H}_{29}^{\cdot }$, and $\hbox {C}_{23}\hbox {H}_{47}\hbox {O}^{\cdot }$).

As the process continues, more complex reactions occur, involving the combination and rearrangement of these fragments to form new compounds ($\hbox {C}_{11}\hbox {H}_{22}$, $\hbox {C}_{19}\hbox {H}_{39}^{\cdot }$, $\hbox {C}_{12}\hbox {H}_{26}$, $\hbox {C}_{8}\hbox {H}_{18}$, and $\hbox {C}_{22}\hbox {H}_{45}^{\cdot }$). Some reactions also involve the addition of hydrogen radicals or removal of water molecules to stabilize newly formed species. Interestingly, some elementary reactions in the Table 3 suggest that PE can revert back to its original form under certain conditions ($\hbox {C}_{18}\hbox {H}_{37}^{\cdot } + \hbox {C}_{12}\hbox {H}_{25}^{\cdot } \rightarrow \hbox {C}_{30}\hbox {H}_{62}$). This observation highlights the reversible nature of some reactions in this system, which may influence the overall efficiency and selectivity of hydrothermal gasification depending on reaction conditions.

**Table 4:** Representative elementary reactions of water and gases in the HTG process obtained by ReaxFF simulations at $\hbox {T} = 1500~\hbox {K}$.

Nr.	Reactions
1	$\hbox {H}_2\hbox {O} \rightarrow \hbox {H}^{\cdot } + \hbox {HO}^{\cdot }$
2	$\hbox {C}_{30}\hbox {H}_{62} + \hbox {HO}^{\cdot } \rightarrow \hbox {C}_{30}\hbox {H}_{61}^{\cdot } + \hbox {H}_2\hbox {O}$

**Table 5:** Representative elementary reactions of water and gases in the HTG process obtained by ReaxFF simulations at $\hbox {T} = 2500\,\hbox {K}$.

Nr.	Reactions
1	$\hbox {H}_2\hbox {O} \rightarrow \hbox {H}^{\cdot } + \hbox {HO}^{\cdot }$
2	$\hbox {C}_{2}\hbox {H}_{4} + \hbox {HO}^{\cdot } \rightarrow \hbox {C}_{2}\hbox {H}_3^{\cdot } + \hbox {H}_2\hbox {O}$
3	$\hbox {C}_{2}\hbox {H}_{4} + \hbox {H}^{\cdot } \rightarrow \hbox {C}_{2}\hbox {H}_{3}^{\cdot } + \hbox {H}_2$
4	$\hbox {C}_{2}\hbox {H}_{4} \rightarrow \hbox {C}_{2}\hbox {H}_{3}^{\cdot } + \hbox {H}^{\cdot }$
5	$\hbox {C}_{3}\hbox {H}_3\hbox {O}^{\cdot } \rightarrow \hbox {C}_{2}\hbox {H}_{3}^{\cdot } + \hbox {CO}$
6	$\hbox {C}_{2}\hbox {H}_{3}\hbox {O}^{\cdot } \rightarrow \hbox {CH}_{3}^{\cdot } + \hbox {CO}$
7	$\hbox {C}_{2}\hbox {H}_{3}^{\cdot } \rightarrow \hbox {C}_{2}\hbox {H}_{2} + \hbox {H}^{\cdot }$
8	$\hbox {C}_{2}\hbox {H}_{3}^{\cdot } + \hbox {H}^{\cdot } \rightarrow \hbox {C}_{2}\hbox {H}_{2} + \hbox {H}_2$
9	$\hbox {C}_{2}\hbox {H}_{2} + \hbox {HO}^{\cdot } \rightarrow \hbox {C}_{2}\hbox {H}_{3}\hbox {O}^{\cdot }$
10	$\hbox {C}_{2}\hbox {H}_{4} + \hbox {C}_2\hbox {H}_2 \rightarrow \hbox {C}_4\hbox {H}_6$
11	$\hbox {C}_{4}\hbox {H}_{6} \rightarrow \hbox {C}_{2}\hbox {H}_{2} + \hbox {C}_{2}\hbox {H}_{4}$
12	$\hbox {C}_{2}\hbox {H}_{4} \rightarrow \hbox {C}_2\hbox {H}_3^{\cdot } + \hbox {H}^{\cdot }$
13	$\hbox {C}_{2}\hbox {H}_{4} + \hbox {H}^{\cdot } \rightarrow \hbox {C}_{2}\hbox {H}_{3}^{\cdot } + \hbox {H}_2$
14	$\hbox {C}_{3}\hbox {H}_{7}^{\cdot } \rightarrow \hbox {C}_{2}\hbox {H}_{4} + \hbox {CH}_3^{\cdot }$
15	$\hbox {C}_{4}\hbox {H}_{9}^{\cdot } \rightarrow \hbox {C}_{2}\hbox {H}_{4} + \hbox {C}_2\hbox {H}_5^{\cdot }$
16	$\hbox {C}_{2}\hbox {H}_{5}^{\cdot } \rightarrow \hbox {C}_{2}\hbox {H}_{4} + \hbox {H}^{\cdot }$
17	$\hbox {C}_3\hbox {H}_6 \rightarrow \hbox {C}_2\hbox {H}_3^{\cdot } + \hbox {CH}_{3}^{\cdot }$
18	$\hbox {C}_3\hbox {H}_7^{\cdot } \rightarrow \hbox {C}_{3}\hbox {H}_6 + \hbox {H}^{\cdot }$
19	$\hbox {C}_{4}\hbox {H}_{9}^{\cdot } \rightarrow \hbox {C}_{3}\hbox {H}_{6} + \hbox {CH}_{3}^{\cdot }$
20	$\hbox {C}_5\hbox {H}_{11}^{\cdot } \rightarrow \hbox {C}_3\hbox {H}_{6} + \hbox {C}_2\hbox {H}_5^{\cdot }$
21	$\hbox {C}_8\hbox {H}_{17}^{\cdot } \rightarrow \hbox {C}_3\hbox {H}_{6} + \hbox {C}_5\hbox {H}_{11}^{\cdot }$
22	$\hbox {C}_2\hbox {H}_5^{\cdot } + \hbox {CH}_3^{\cdot } \rightarrow \hbox {C}_{3}\hbox {H}_8$
23	$\hbox {C}_3\hbox {H}_8 \rightarrow \hbox {C}_3\hbox {H}_7^{\cdot } + \hbox {H}^{\cdot }$
24	$\hbox {C}_3\hbox {H}_8 + \hbox {H}^{\cdot } \rightarrow \hbox {C}_3\hbox {H}_7^{\cdot } + \hbox {H}_2$
25	$\hbox {C}_4\hbox {H}_9^{\cdot } + \hbox {H}^{\cdot } \rightarrow \hbox {C}_4\hbox {H}_{10}$

The representative elementary reactions involving water and gas molecules at a temperature of 1500 K is illustrated in Table 4. It is worth mentioning that in these specific simulations, no reaction pathways for gas formation were observed. This observation suggests that the temperature of 1500 K may be insufficient to promote gas formation. The Table 5 presents a set of representative elementary reactions involved in water and gases formation during the HTG process at a higher temperature 2500 K. The gas formation process typically involves multiple steps. The breakdown of larger hydrocarbons primarily leads to the formation of smaller fragments like radicals ($\hbox {C}_3\hbox {H}_7^{\cdot }$, $\hbox {C}_4\hbox {H}_9^{\cdot }$, $\hbox {H}^{\cdot }$, $\hbox {HO}^{\cdot }$, etc), alkenes, and other species such as water vapor and carbon monoxide.

The initial reaction between hydrocarbon molecules and water or radical species result in the formation of new intermediate products. For instance, $\hbox {C}_2\hbox {H}_2$ reacts with a hydroxyl radical ($\hbox {HO}^{\cdot }$) to produce an alkoxy radical ($\hbox {C}_2\hbox {H}_3\hbox {O}^{\cdot }$), which can further decompose into smaller fragments like carbon monoxide and methyl radicals (CO, $\hbox {CH}_3^{\cdot }$). Similar reactions occur for other hydrocarbon molecules, leading to the formation of various gaseous compounds. As the process continues, these intermediate products react with each other or undergo further transformations, resulting in the formation of additional gas species. For example, $\hbox {C}_2\hbox {H}_4$ and $\hbox {C}_2\hbox {H}_2$ can combine to form a larger hydrocarbon molecule ($\hbox {C}_4\hbox {H}_6$).

The elementary reactions involved in the HTG process, as depicted in Table 5, reveal a prominent role for hydrogen ($\hbox {H}^{\cdot }$) and hydrox ($\hbox {HO}^{\cdot }$) radicals. These reactive species are key players in shaping the reaction mechanisms, participating in elementary reactions such as abstraction, combination, and decomposition. The significance of H radicals is particularly evident in reactions where they facilitate the transfer of hydrogen radicals between molecules (see Table 5 for reactions 3, 8, 13, 18, and 24). This process enables the formation of new chemical bonds, thereby influencing the reactivity of molecules. In addition, the participation of $\hbox {HO}^{\cdot }$ radicals in radical combination and hydrogen abstraction reactions underscores their importance in HTG. The prevalence of both hydrogen and hydroxyl radicals highlights their critical roles in promoting gas formation during HTG. These reactive intermediates play a crucial part in facilitating chemical transformations and ultimately influencing the composition of the gaseous products formed.

### Product formation at different temperatures

**Figure 3:**
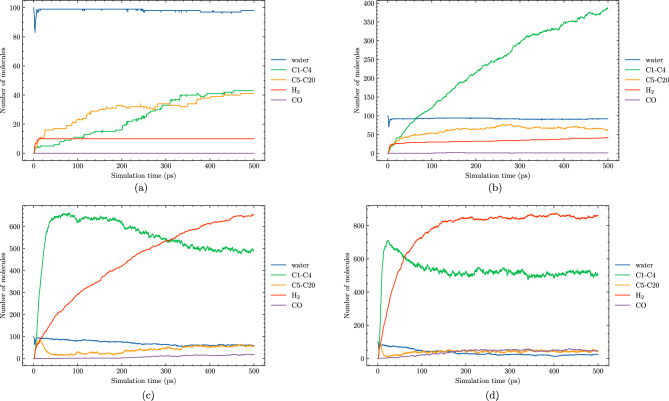
Molecule evolution during the HTG process of system 1 at different temperatures of 1700 K (**a**), 2100 K (**b**), 3000 K (**c**) and 3600 K (**d**).

In this section, we explore the effects of temperature on the hydrothermal decomposition of polyethylene microplastics and elucidate how variations in temperature influence product composition. Our findings, as depicted in Fig. 3, demonstrate that at lower temperatures, such as 1700 K, the degradation process is marked by limited gas production and the predominance of larger hydrocarbon molecules (C5–C20). This observation suggests that at these temperatures, polyethylene microplastics experience minimal structural degradation, with much of the original polymer chain intact. With the increase in temperature to 2100 K, we observe a substantial enhancement in the formation of smaller hydrocarbon species (C1–C4). This is attributed to elevated reaction rates and more efficient molecular fragmentation due to higher thermal energy inputs, leading to extensive degradation and gasification.

**Figure 4:**
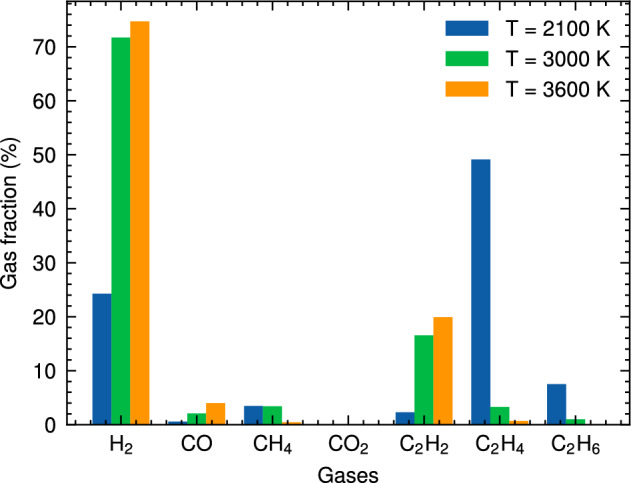
Gas fraction composition of the HTG process for system 1 at different temperatures.

It is crucial to acknowledge that our molecular dynamics (MD) simulations are limited to nanosecond time scales due to computational constraints. As a result, the observed degradation of PE during the hydrothermal gasification process might be underestimated in our simulations, as the full extent of structural degradation may occur beyond the simulated time frame. In real experiments, however, hydrothermal gasification at 1700 K is typically conducted at temperatures far exceeding those accessible in MD simulations and for much longer reaction times (minutes or hours). These experimental conditions provide sufficient time and energy for PE degradation to take place, leading to the observed gasification process^15^.

We observe that at $\hbox {T} = 2100 \,\hbox {K}$, the decomposition process favor the formation of smaller hydrocarbon molecules (C1–C4). In contrast, as temperature increases beyond this point, the reaction mechanisms involved in HTG undergo significant changes to more hydrogen molecule. One possible explanation for the increased formation of $\hbox {H}_2$ at higher temperatures is the enhanced reactivity of the PE microplastic matrix with hydrogen and hydroxyl radicals. As the thermal energy available for bond breakage and rearrangement increases, C1–C4 reaction more extensively with these reactive species to form hydrogen gas molecules. Consistently, our simulations indicate that the number of $\hbox {H}_2$ formed is significantly higher at temperatures above 3000 K compared to lower temperatures. This is in excellent agreement with experiment data by Cao et al.^15^. Their study also showed that the yield of $\hbox {H}_2$ gas increases significantly at higher temperatures^15^, further validating our findings.

Figure 4 provides a detailed breakdown of the gas fraction composition for the major gaseous products generated during HTG of polyethylene microplastics. The data presented in this figure reveal that $\hbox {H}_2$, CO, $\hbox {CO}_2$, $\hbox {CH}_4$, and C2 are responsible for more than 90% of the total gas produced under these conditions. It is noteworthy that hydrocarbon species with carbon chains containing three (C3) or four (C4) carbons are present in very small quantities, suggesting that longer polymer chains are less likely to be preserved during the degradation process. This observation further implies that HTG effectively breaks down PE microplastics into smaller fragments and gaseous compounds through multiple reaction pathways.

As demonstrated in Fig. 4, $\hbox {H}_2$ is indeed the most abundant gas species generated during the HTG process, followed by $\hbox {C}_2\hbox {H}_4$ and $\hbox {C}_2\hbox {H}_{6}$. The presence of small amounts of CO and $\hbox {CH}_4$ indicates that these gases are also formed as a result of polyethylene decomposition under supercritical water conditions. The temperature dependence of the gas fraction composition is particularly notable. For example, at $\hbox {T} = 2100 \,\hbox {K}$, the gas fraction of $\hbox {H}_2$ is only approximately 25%, while at higher temperatures (e.g., $\hbox {T} = 3600 \,\hbox {K}$), it increases dramatically to around 75%. This significant increase in hydrogen production with increasing temperature can be attributed to several factors, including enhanced reaction rates and increased molecular fragmentation propensities at elevated thermal energy inputs.

Notably, Fig. 4 reveals an absence of $\hbox {CO}_2$ across all cases studied, implying that this gas is not a significant contributor to the HTG process for polyethylene microplastics in our simulations. This observation can be attributed to the unique characteristics of the HTG process, where supercritical water acts as reactant. Unlike traditional thermal decomposition processes that rely on oxygen-rich environments for combustion, supercritical water enables PE to degrade in the presence of hydrogen and hydroxyl radicals, which are generated from dissociated water molecules. These radicals play a crucial role in breaking C–C and C–H bonds within the polymer chain, leading to the formation of hydrogen, CO, C2, and other light organic compounds. The absence of CO2 in our simulations is therefore consistent with the HTG conditions and the critical role played by supercritical water in this process. The results demonstrate a dominant formation of C2 species throughout the entire temperature range examined, consistent with previous work^35^.

**Figure 5:**
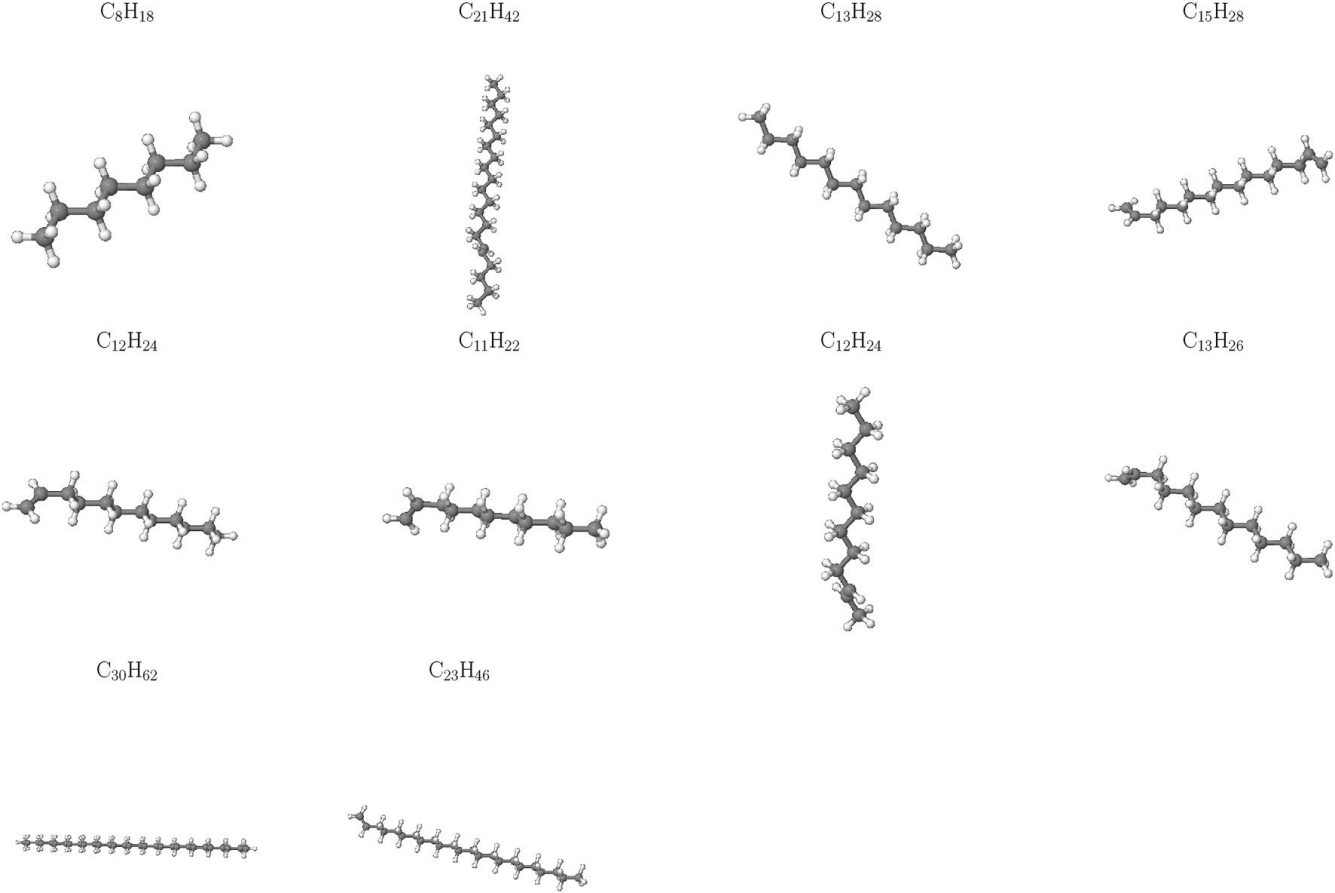
Snapshot of the largest product molecules with their chemical formula during the HTG process of system 1 at temperature at 2100 K. The gray and white color represent for C, and H atoms, respectively.

**Figure 6:**
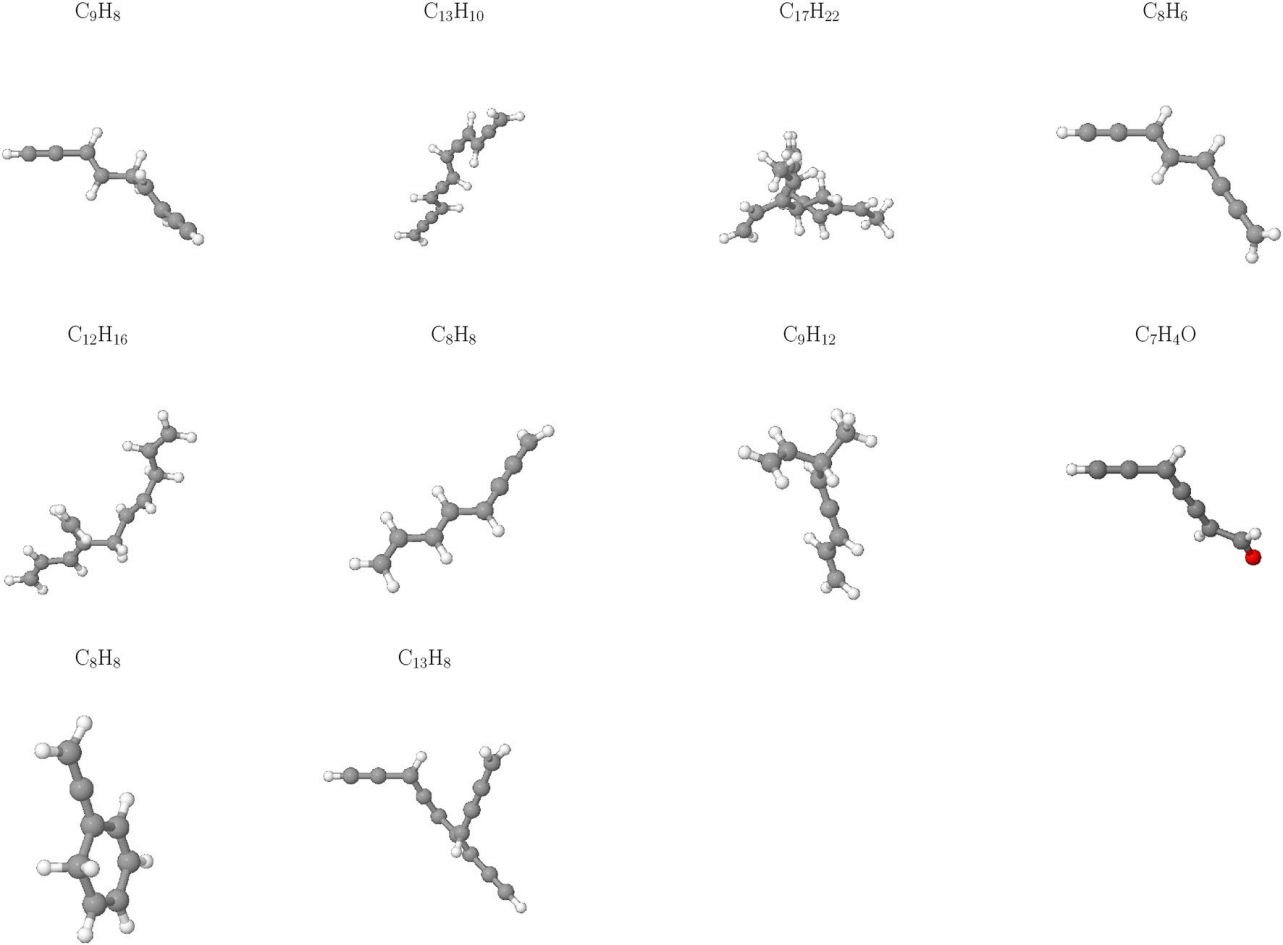
Snapshot of the largest product molecules with their chemical formula during the HTG process of system 1 at temperature at 3200 K. The gray, white and red color represent for C, H and O atoms, respectively.

The influence of temperature on the largest product formation during HTG of polyethylene microplastics was investigated. At $\hbox {T} = 2100 \,\hbox {K}$ (Fig. 5), the decomposition process is observed to be relatively slow and incomplete, with larger fragments of PE, such as $\hbox {C}_{21}\hbox {H}_{42}$ and $\hbox {C}_{12}\hbox {H}_{24}$, remaining intact in the largest product distribution. This suggests that under these conditions, further increases in temperature may be necessary to promote more extensive degradation and gasification of polyethylene microplastics.

In contrast, at $\hbox {T} = 3200 \,\hbox {K}$ (Fig. 6), a significant increase in the formation of smaller hydrocarbon species ($\hbox {C}_1$–$\hbox {C}_4$) as well as more complex organic molecules containing benzene rings (e.g., $\hbox {C}_8\hbox {H}_8$) or oxygen atoms (e.g., $\hbox {C}_7\hbox {H}_4\hbox {O}$) is observed. This indicates that at higher temperatures, the largest product tends to form more complex organic compounds, which may have distinct environmental fates and impacts compared to simpler hydrocarbon molecules like ethylene or ethane. The formation of these complex organic compounds may be attributed to the increased thermal energy available at elevated temperatures, enabling the breakdown of PE into smaller fragments that can then react with each other to form more intricate molecular structures. This phenomenon underscores the significance of temperature as a parameter in governing chemical pathways and product distributions during the HTG process of polyethylene microplastics.

**Figure 7:**
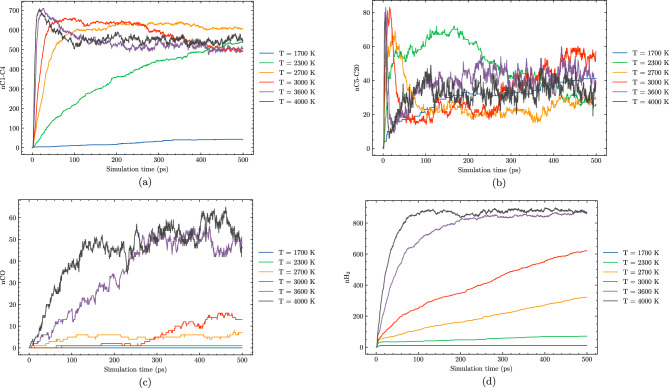
Temperature-dependent evolution of major products during HTG of system 1.

Figure 7 provides a comprehensive overview of the temperature-dependent evolution of major products during hydrothermal gasification of polyethylene microplastics. Each subplot focuses on one specific species, allowing for a clearer comparison of its concentration as a function of temperature. The figure reveals that the formation of C1–C4 species reaches its maximum at $\hbox {T} = 2700\,\hbox {K}$, before gradually decreasing as temperature continues to rise above this point (Fig. 7). This observation is interesting, as it suggests that under these conditions, polymerization reactions may be occurring, leading to the formation of CO, larger hydrocarbon molecules (C5–C20) and potentially more complex organic compounds. In addition to the peak in C1–C4 species, Fig. 7 demonstrates that the amount of C5–C20 species remains relatively small across all temperature ranges studied.

One interesting feature of Fig. 7 is the pronounced peak in $\hbox {H}_2$ production at around 3600 K, which gradually decrease at higher temperatures. This phenomenon can be attributed to the delicate balance between reactant concentrations and reaction rates under supercritical water conditions, facilitating efficient degradation of polyethylene microplastics and a sufficient supply of carbon atoms as reactants. As the temperature approaches this optimal range, the reaction rate likely slows down enough to establish an equilibrium state in which hydrogen formation occurs efficiently. In contrast, when temperatures exceed 3600 K, the hydrogenation reactions might occur, resulting in the preferential formation of larger hydrocarbon molecules and complex organic compounds. This reduction in reactant availability can subsequently cause the yield of $\hbox {H}_2$ to plateau.

The observation that hydrogen production from HTG begins to plateau and ultimately decreases at temperatures above 3600 K suggests that optimal conditions for $\hbox {H}_2$ production are narrowly defined, and exceeding these temperatures can lead to a negative effect on reaction outcomes. Too high temperature in HTG can result in uncontrolled and more hydrogenation, leading to the formation of larger hydrocarbon molecules and complex organic compounds. This not only reduces the availability of carbon atoms as reactants for $\hbox {H}_2$ production but also competes with hydrogen formation reactions, ultimately hindering the overall yield of this valuable product. In light of these findings, it is essential to optimize HTG conditions within a optimal temperature range to ensure efficient and high-yielding hydrogen production while minimizing the formation of undesirable byproducts.

In gasification simulations, it is commonly observed that the simulated temperature is typically two to three times higher than the experimental temperature^35^. This discrepancy arises due to the inherent challenges in establishing a direct correlation between simulation and experiment, as well as differences in the thermal conditions experienced by the system during the computational modeling process compared to real-world scenarios. To establish accurate correlations between the two, further experimental studies are required to develop equations that can convert simulation temperatures to more realistic experimental conditions. This area of research remains an important topic for future investigation.

### Effect of water content

HTG processes rely heavily on the presence of water as an effective medium for heat transfer and a promoter of reactivity in the decomposition of polyethylene microplastics. In this section, we will investigate the impact of varying water content on HTG reactions and its subsequent product formation dynamics.

**Figure 8:**
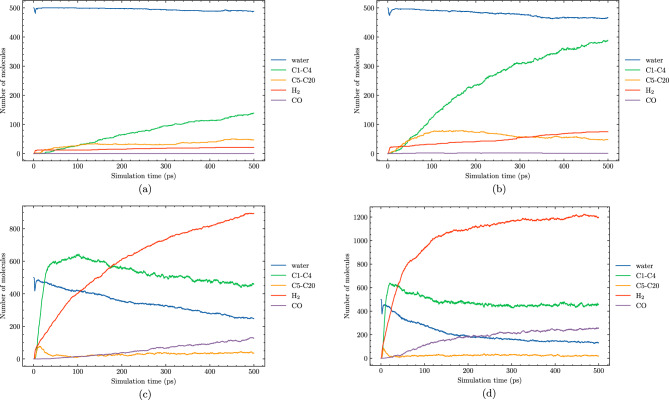
Molecule evolution during the HTG process of system 3 at different temperatures of 1900 K (**a**), 2100 K (**b**), 3000 K (**c**) and 3600 K (**d**).

**Figure 9:**
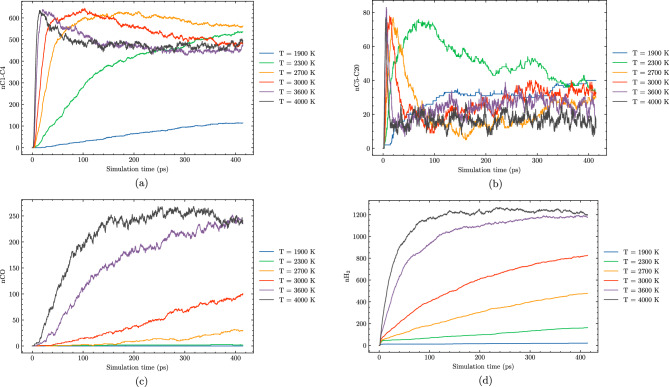
Temperature-dependent evolution of major products during HTG of system 3.

Figures 8 and 9 offer valuable insights into the effects of different water contents on product formation during HTG processes. System 3, featured in these figures, has a 30% water content compared to system 1’s 8% water content. By comparing these two systems, we can gain a better understanding of how changes in water content influence reaction outcomes and identify potential trends for optimizing reaction conditions.

At lower temperatures, such as $\hbox {T} = 1900\,\hbox {K}$ and $\hbox {T} = 2100 \,\hbox {K}$, both system 1 and system 3 exhibit similar behavior in terms of product formation, implying that the water content has a negligible impact on the overall gasification process under these conditions. Furthermore, the conversion rate is remarkably low at these temperatures.

In contrast, as the temperature increases to $\hbox {T} = 3000 \,\hbox {K}$ and beyond, a significant difference in the hydrogen production between system 1 and system 3. For instance, at $\hbox {T} = 3600 \,\hbox {K}$, the amount of $\hbox {H}_2$ generated in system 3 is almost 50% higher compared to that observed in system 1. This notable observation can be attributed to the enhanced formation of hydroxyl and H radical species in the presence of higher water contents, which are well-known to promote more extensive decomposition pathways during HTG processes. The increased production of these radical species facilitates a greater extent of polyethylene microplastic degradation, leading to the generation of a larger quantity of $\hbox {H}_2$. The observations suggest that increasing the water content in system 3 promotes a more favorable reaction environment for HTG, resulting in improved hydrogen yields at elevated temperatures.

Interestingly, as depicted in Fig. 8, at elevated temperatures (i.e., 3000 K and 3600 K), the number of C1–C4 species reaches a peak before slightly decreasing. This phenomenon can be attributed to the fact that at higher temperatures, C1–C4 molecules undergo reactions that convert them into CO or higher oligomeric species (C5–C20). The Supplementary Information (SI) provides additional insights by presenting a plot of total carbon content, which clearly demonstrates that as the temperature increases, the number of carbon atoms in C1–C4 molecules decreases simultaneously with an increase in the number of carbon atoms in CO and C5–C20 species.

**Figure 10:**
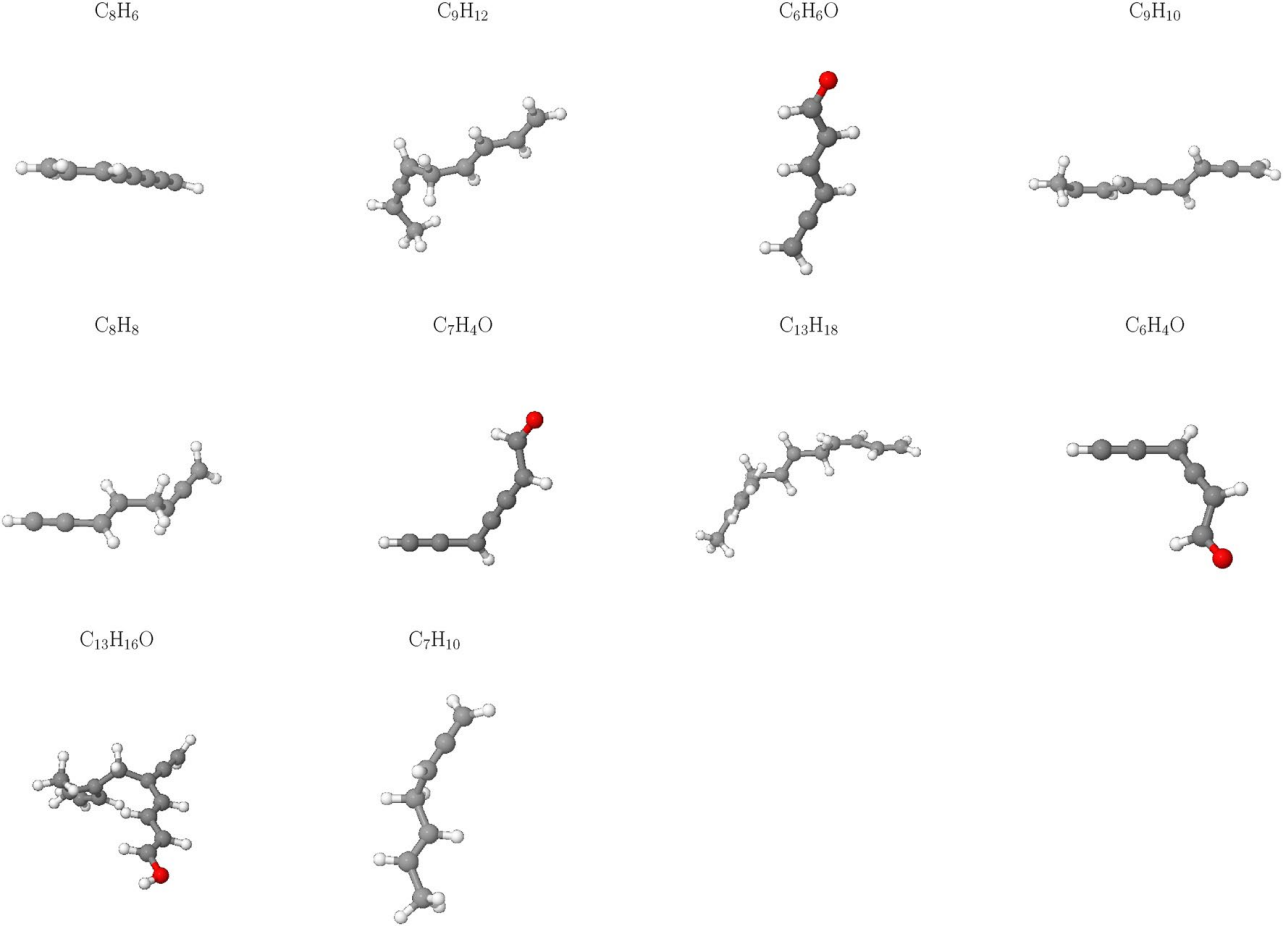
Snapshot of the largest product molecules with their chemical formula during the HTG process of system 3 at temperature at 3200 K. The gray, white and red color represent for C, H and O atoms, respectively.

To investigate further the effects of varying water content on the formation of the larger products during HTG processes, a comparative analysis was conducted between the largest products obtained from HTG of system 3 and system 1 at $\hbox {T} = 3200 \,\hbox {K}$. Figures 6 and 10 reveal striking differences between the two systems with regards to product composition and complexity. In particular, system 3, which has a higher water content, appears to generate a greater diversity of organic compounds containing oxygen atoms, such as $\hbox {C}_6\hbox {H}_6\hbox {O}$, $\hbox {C}_6\hbox {H}_4\hbox {O}$, $\hbox {C}_7\hbox {H}_4\hbox {O}$, and $\hbox {C}_{13}\hbox {H}_{16}\hbox {O}$.

The increased water content during the HTG process in system 3 likely promotes the formation of more complex organic species by facilitating the degradation pathways of PE. As a result, the reaction conditions in system 3 may lead to the generation of a broader range of oxygenated compounds, which could have implications for environmental remediation strategies and carbon cycling. The difference in product composition between system 1 and system 3 highlights the critical role that water content plays in shaping the HTG process.

**Figure 11:**
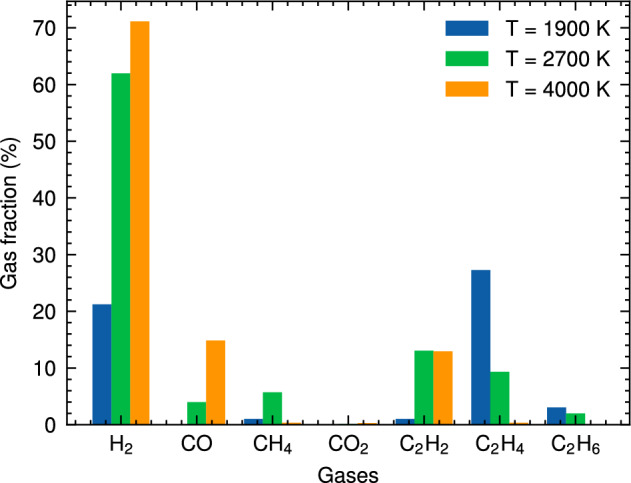
Gas fraction composition of the HTG process for system 3 at different temperatures.

The gas fraction composition of major gaseous products generated during HTG provides valuable insights, as demonstrated in Fig. 11. With a focus on system 3, which boasts a higher water content compared to system 1, the data reveal that $\hbox {H}_2$ remains the most abundant gas species across all temperature ranges studied, followed by $\hbox {C}_2\hbox {H}_4$ and $\hbox {C}_2\hbox {H}_{6}$. A closer examination of Fig. 11 shows significant variations in hydrogen fractions between systems 1 and 3 under different temperature conditions. At $\hbox {T} = 2700 \,\hbox {K}$, system 3 exhibits a notably higher hydrogen fraction, approximately 62%, whereas system 1 displays a relatively lower value of 52%. This difference suggests that more hydrogen production in HTG with higher water content.

However, as the temperature increases to $\hbox {T} = 4000 \,\hbox {K}$, an interesting trend was observed. The $\hbox {H}_2$ fractions for both systems approach a similar maximum value of around 70% as the hydrothermal gasification process becomes saturated. Furthermore, a thorough examination of Fig. 11 reveals striking differences in the proportions of $\hbox {C}_2\hbox {H}_4$ and $\hbox {C}_2\hbox {H}_{6}$ between the two systems. While system 1 tends to produce a larger proportion of $\hbox {C}_2\hbox {H}_4$ at lower temperatures, system 3 exhibits an increase in $\hbox {C}_2\hbox {H}_{6}$ production as temperature rises. These observation may be attributed to the fact that at high water content, the increased availability of H radicals and hydrogenation reactions favor the conversion of $\hbox {C}_2\hbox {H}_4$ into $\hbox {C}_2\hbox {H}_{6}$. This reaction path is enhanced under conditions with abundant water molecules, which act as sources of H atoms and facilitate the hydrogenation process. As a result, higher water content can lead to a shift in product distribution towards more ethane formation.

### Kinetic and activation energy

Understanding the reaction kinetics of the HTG process is vital for grasping how efficiently reactants are converted into products. This knowledge is crucial in optimizing the HTG conditions to achieve maximum yields and minimize reaction times. A widely used approach in HTG research has been to analyze the carbon conversion rate^35,40^, which provides valuable insights into the kinetic behavior of the system.

Equation (2) defines the carbon conversion (CC) as a percentage, calculated by dividing the sum of the number of CO, $\hbox {CO}_2$, and CmHn gaseous molecules produced during hydrothermal gasification by the initial number of carbon atoms in the PE, $n_0$.

2 $$\begin{aligned} \text {Carbon conversion } (\%) = \frac{n_{\hbox {CO}} + n_{{\text{CO}}_2} + n_{{{\text{CmHn}}}}}{n_0} \times 100\%. \end{aligned}$$

In this equation, $n_{{{\text{CO}}}},$ $n_{{{\text{CO}}_2}}$, and $n_{{\text{C}}_m{{\text{H}}}_n}$ represent the number of molecules of carbon monoxide, carbon dioxide, and other hydrocarbon gaseous, respectively. The variable $n_0$ denotes the initial number of carbon atoms present in PE.

One of the key parameters that can be extracted from the carbon conversion rate data is the activation energy (Ea) of the HTG process. We will explore the Ea values obtained from the ReaxFF simulations. By continuously monitoring the carbon conversion rate as a function of time, enabled us to determine the reaction rate and how it varied in response to different. To analyze this dependence, we employed the rate law of first-order reaction equation, which is depicted by Eq. (3).

3 $$\begin{aligned} \frac{\text {d}[A]}{\text {d}t} = -k[A]. \end{aligned}$$

In this equation, [A] represents the carbon conversion rate in the system at any given time t, and k is the rate constant that governs the reaction’s kinetics.

By fitting the data to the integral form of Eq. (3), we were able to determine the value of k for each temperature condition, thereby enabling us to assess the effects of temperature on the reaction rate. The obtained results allowed us to draw valuable insights into the mechanisms underlying the hydrothermal gasification process and its dependence on temperature.

The rate constant k in Eq. (3) obeys the Arrhenius relationship presented in Eq. (4).

4 $$\begin{aligned} k = Ae^{-\frac{Ea}{RT}}. \end{aligned}$$

In Eq. (4), Ea is the activation energy, R is the gas constant and T is the absolute temperature.

**Figure 12:**
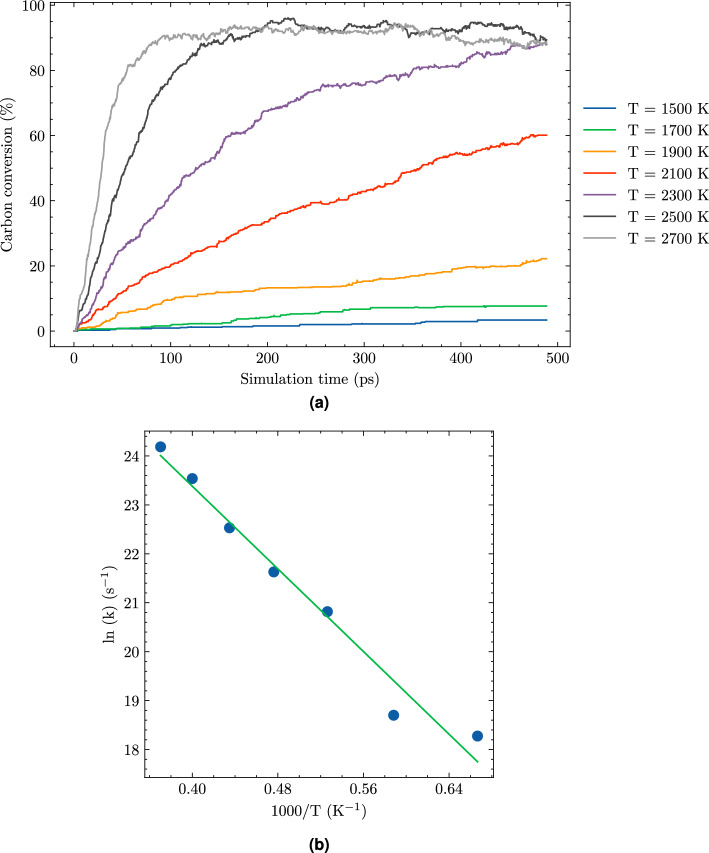
Carbon conversion of system 1 during HTG process at different temperatures (**a**) and the plot of logarithm of rate constant as a function of the inverse temperatures (**b**).

Figure 12a presents an illustration of the carbon conversion rates for system 1 at various temperatures, highlighting the significant impact of temperature on this critical parameter. At a relatively low temperature of 1500 K, minimal reaction is observed, with a carbon conversion rate of approximately 1%. As the temperature increases, so does the rate of carbon conversion. At a higher temperature of 2300 K, the maximum conversion rate of 90% is achieved after a simulation time of 500 picoseconds (ps).

However, it is essential to note that the required time for reaching this maximum value decreases significantly with increasing temperatures. For instance, at an even higher temperature of 2500 K, the carbon conversion reaches its peak within 150 ps, while at temperature of 2700 K, this peak is achieved in just 50 ps. These findings underscore the significance of temperature in facilitating the HTG process.

**Table 6:** Activation energy (Ea) of the HTG process of microplastics PE obtained from ReaxFF MD.

System	Water content	Ea (kJ/mol)
1	8%	$176 \pm 13$
2	20%	$183 \pm 12$
3	30%	$213 \pm 5$
4	37%	$268 \pm 8$
Thermal gasification^35,40^	–	340–344

Figure 12b exhibits a plot of the natural logarithm of the rate constant (ln(k)) against the reciprocal of temperature (1/T) for system 1, which demonstrates a strong linear correlation. This linear dependence is consistent with the expected behavior of an Arrhenius relationship. From this analysis, we derive an activation energy (Ea) of 176 kJ/mol for system 1.

The Table 6 presents a compilation of Ea for the HTG process of microplastics PE, obtained through ReaxFF MD for all systems studied with varying water content. The results show that the activation energy increases as the water content in the system increases. For instance, system 1, with 8% water content, has an Ea of $176 \pm 13$ kJ/mol, while System 4, with 37% water content, has an Ea of $268 \pm 8$ kJ/mol.

This observed increase in Ea with rising water content is consistent with previous studies on the HTC process^30^. According to these investigations, water molecules participate actively in the reaction mechanism, necessitating their decomposition into radicals for efficient reactivity. Furthermore, the increase in water content can also hinder the diffusion of reactants within the system, contributing to the elevated Ea values^30^. These findings underscore the critical role of water content in determining the HTG process’s activation energy.

To the best of our knowledge, there is no previously published activation energy value specifically for the HTG process of PE. In light of this, we have chosen to compare our results with those obtained through thermal gasification (TG) studies^35,40^. This comparison provides a valuable insight into the relative energies required for microplastic degradation via these two distinct processes. Interestingly, our ReaxFF simulations reveal significantly lower activation energies for the HTG process compared to those reported in TG. Previous computational and experimental studies^35,40^ have documented activation energy values ranging from 340 to 344 kJ/mol for TG, which is roughly 100–150 kJ/mol higher than the Ea values obtained through our HTG simulations. This notable difference can be attributed primarily to the unique role of water in the HTG process. In contrast to TG, where water is absent or plays a minimal role, water serves as a reactant in the HTG process. It promotes degradation and gasification reactions by providing hydrogen and hydroxyl radicals. These highly reactive species enhance the breakdown of PE chains and facilitate the conversion of PE to syngas, ultimately leading to lower activation energies in HTG compared to TG processes.

The results highlight the potential advantages of employing the HTG process for microplastic degradation. One such benefit is the reduced energy requirements necessary to achieve efficient decomposition. This could have significant implications for industrial-scale microplastic processing, particularly in scenarios where energy efficiency is a major concern.

The presence of water in HTG systems has a profound impact on two crucial parameters: hydrogen production rates and activation energies. The optimal balance of these factors is essential for achieving efficient plastic waste degradation while minimizing energy consumption and costs. As the water content in HTG systems increases, both hydrogen production rates and activation energies are affected. Higher water concentrations have been shown to enhance hydrogen yields due to the increased availability of reactants and the favorable reaction conditions. However, this increase in water content also leads to higher activation energies. Activation energy represents the minimum energy required for a chemical reaction to proceed, and increasing water content necessitates greater energy input to overcome the higher energy barriers associated with these systems.

The key to achieving efficient plastic waste degradation while minimizing energy consumption and costs lies in finding the optimal water concentration. This balance point must be carefully tuned to maximize hydrogen yields while minimizing the energy required to overcome activation energies. The identification of this optimal water concentration is critical for scaling up HTG processes to industrial levels, where efficiency, cost-effectiveness, and environmental sustainability are important.

## Conclusions

This work provides an in-depth analysis of the hydrothermal gasification (HTG) process for polyethylene (PE), a key component in the growing issue of microplastics pollution. Utilizing ReaxFF molecular dynamics simulations, our study has shed light on the complex interplay of factors that influence the HTG process, including temperature, water content, carbon conversion rate, and product yields. The simulation data not only validate the efficacy of ReaxFF MD in modeling such reactions but also confirm that $\hbox {H}_2$, CO and C2 molecules are the principal products obtained from the gasification of PE, aligning with previous findings. This discovery paves the way for innovative strategies to manage microplastic waste sustainably.

Our results demonstrate that temperature plays a pivotal role in enhancing the efficiency of HTG. As temperatures rise, we observe an increase in carbon conversion efficiency up to 90% and peak hydrogen yield of approximately 70%, due to improved bond breaking and formation dynamics. Conversely, water content has a dual effect on HTG: it significantly boosts hydrogen production, yet simultaneously raises the activation energy barrier for the reaction. This finding underscores the need for precise control over process conditions to maximize performance and efficiency. The observed activation energies of 176–268 kJ/mol are notably lower than those associated with traditional thermal gasification (TG), suggesting that HTG is a more efficient method for converting PE into hydrogen-rich syngas.

The implications of this research highlight the potential for future investigations into catalytic systems and co-hydrothermal processes involving biomass. Such advancements could further diminish the activation energy required for PE gasification, offering additional avenues to mitigate plastic pollution. The efficient conversion of waste plastics into useful chemical products is essential for reducing environmental impact and fostering a circular economy.

In conclusion, our study underscores the value of molecular dynamics simulations in unraveling complex hydrothermal processes. The insights gained from ReaxFF MD offer a foundation for developing advanced technologies capable of addressing the pressing challenges of plastic waste management. By harnessing the power of HTG, we can unlock new pathways for sustainable resource utilization and contribute to a future where environmental sustainability is at the forefront of technological innovation. The findings of this research provide a compelling argument for continued exploration and optimization of HTG as a key technology in the fight against microplastic pollution.

### Supplementary Information

Supplementary Information.

## Data Availability

The datasets generated and/or analyzed during the current study are available from the corresponding author on reasonable request.
