# Supplementary material for: Insights into hydro thermal gasification process of microplastic polyethylene via reactive molecular dynamics simulations
Source: Sci Rep. 2024 Aug 13;14:18771. doi: 10.1038/s41598-024-69337-z (PMC11322303; doi:10.1038/s41598-024-69337-z)
Supplement: Supplementary file 1 — Supplementary Information. [file 41598_2024_69337_MOESM1_ESM.pdf]

Supporting Information: Insights into hydro thermal gasification process of microplastic Polyethylene via reactive molecular dynamics simulations

Do Tuong Ha,[*,†] Hien Duy Tong,[‡] and Thuat T. Trinh[*,¶]

†*Faculty of Applied Sciences, Ton Duc Thang University, Ho Chi Minh city, Vietnam*

‡*Faculty of Engineering, Vietnamese-German University (VGU), Thu Dau Mot City, Binh Duong Province, Vietnam*

¶*Porelab, Department of Chemistry, Norwegian University of Science and Technology, Høgskoleringen 5, 7491-Trondheim, Norway*

E-mail: dotuongha@tdtu.edu.vn; thuat.trinh@ntnu.no

This Supplementary Information (SI) offers enhanced insights into various aspects of the hydrothermal gasification process. It includes additional snapshots capturing system dynamics, thorough analysis of product formation across diverse systems, detailed investigation of gas fraction variations with temperature, comprehensive examination of carbon content in distinct products, and supplementary plots illustrating carbon conversion and kinetics under varying water contents.

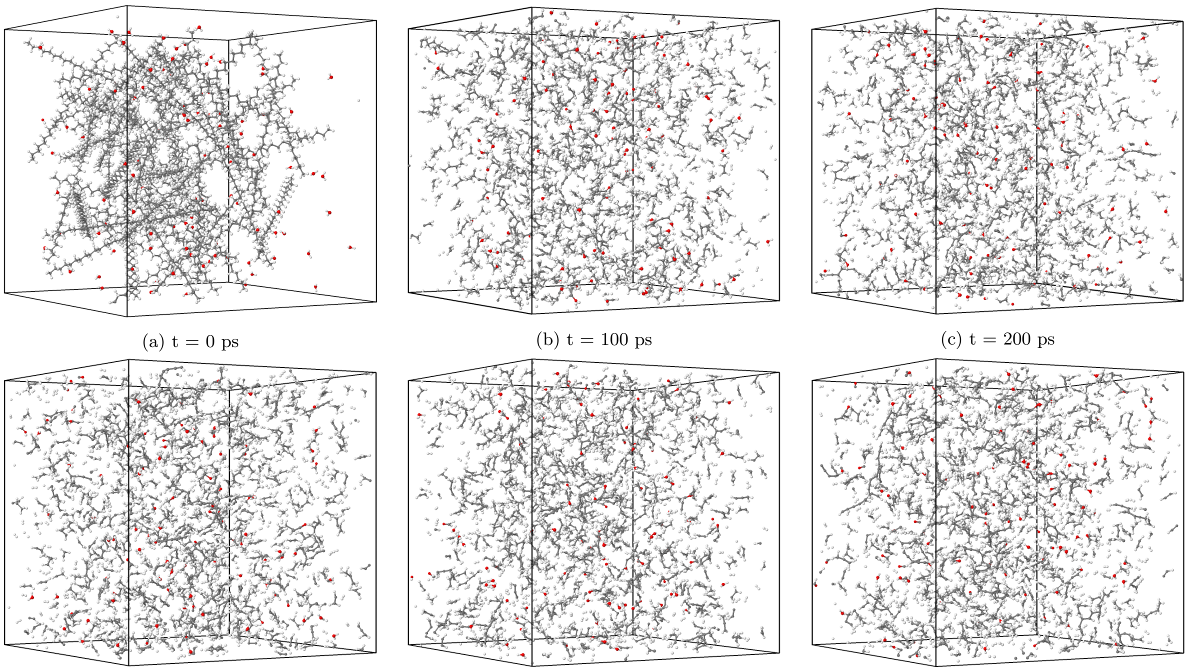

(a) t = 0 ps (b) t = 100 ps (c) t = 200 ps

(d) t = 300 ps (e) t = 400 ps (f) t = 500 ps

Figure S1: Selected snapshots of the HTG process of PE (system 1) at T = 3000K.

Table S1: Gas fraction composition of the HTG process of PE for system 1 at different temperatures.

Gases	1700K	1900K	2100K	2300K	2500K	2700K	3000K	3200K	3400K	3600K	3800K	4000K	4200K
H2	34.5	27.5	24.3	20.3	34.9	52.5	71.7	74.7	73.6	74.7	73.4	74.2	73.8
CO	0.0	0.0	0.6	0.2	0.5	1.0	2.1	3.1	4.1	4.0	4.5	4.0	4.9
CH4	6.9	0.0	3.5	4.7	7.3	8.6	3.4	1.7	0.6	0.4	0.4	0.4	0.2
CO2	0.0	0.0	0.0	0.0	0.0	0.0	0.0	0.0	0.0	0.0	0.0	0.2	0.0
C2H2	0.0	1.7	2.3	3.7	7.2	11.5	16.6	17.8	19.8	19.9	19.7	20.3	20.2
C2H4	17.2	20.8	49.1	32.7	25.6	13.5	3.3	1.2	1.0	0.7	1.2	0.9	0.4
C2H6	0.0	4.2	7.5	7.9	8.2	5.8	1.0	0.1	0.0	0.0	0.0	0.0	0.0
C3H4	0.0	3.3	4.6	5.4	6.1	4.4	2.0	1.5	1.0	0.3	0.7	0.0	0.4
C3H6	37.9	35.0	0.0	17.6	5.9	2.0	0.0	0.0	0.0	0.0	0.0	0.0	0.1
C3H8	0.0	1.7	6.9	6.9	3.9	0.7	0.0	0.0	0.0	0.0	0.0	0.0	0.0
C4H6	0.0	0.8	0.0	0.0	0.0	0.0	0.0	0.0	0.0	0.0	0.0	0.0	0.0
C4H8	0.0	1.7	0.0	0.0	0.0	0.0	0.0	0.0	0.0	0.0	0.0	0.0	0.0
C4H10	3.4	3.3	1.2	0.5	0.2	0.0	0.0	0.0	0.0	0.0	0.0	0.0	0.0

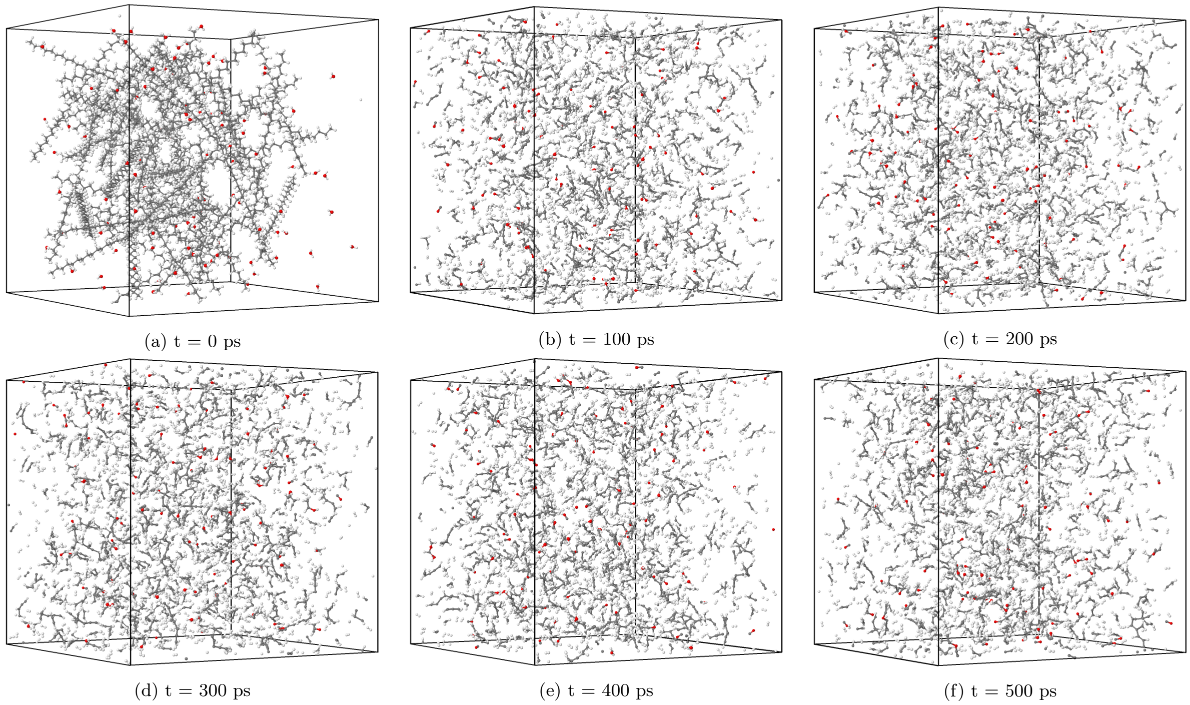

Figure S2: Selected snapshots of the HTG process of PE (system 1) at T = 4000K.

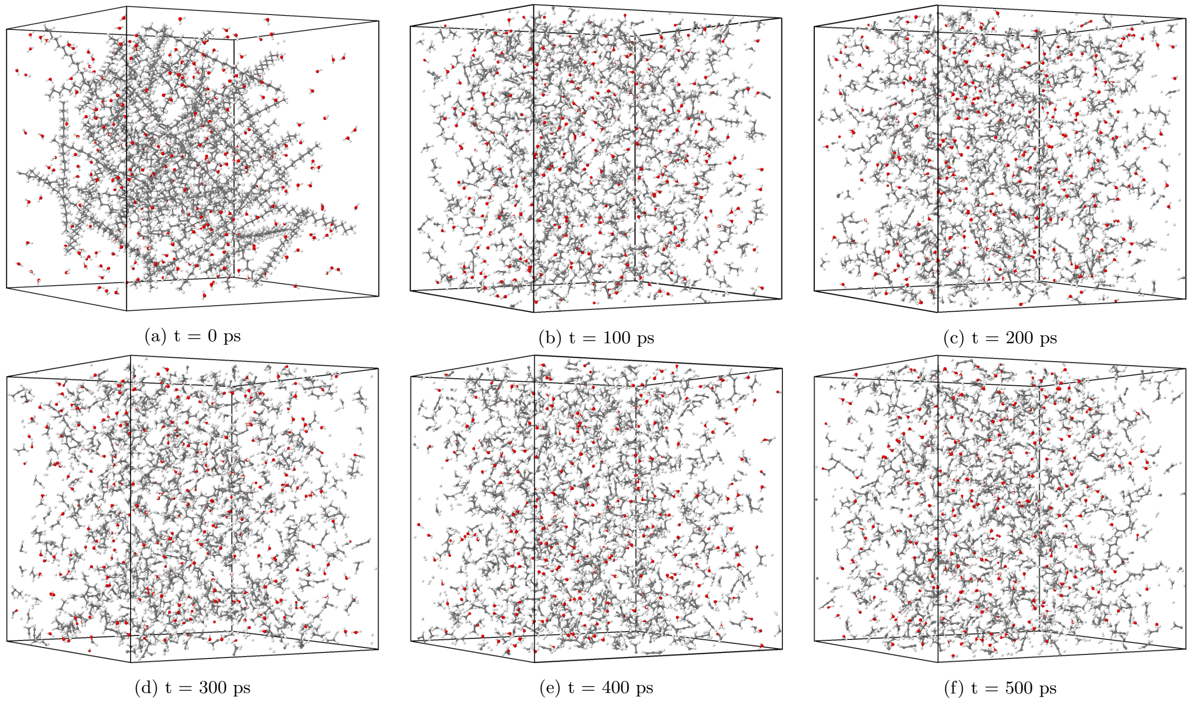

Figure S3: Selected snapshots of the HTG process of PE (system 2) at T = 2500K.

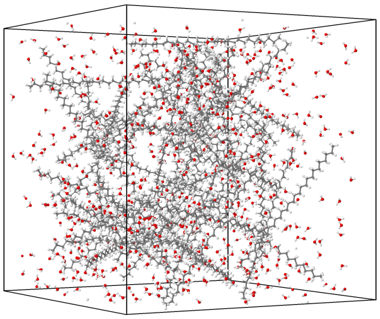

(a) t = 0 ps

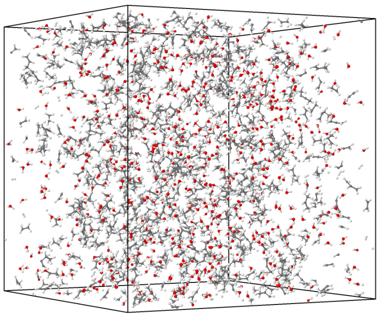

(b) t = 100 ps

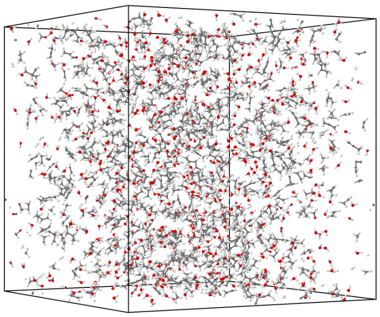

(c) t = 200 ps

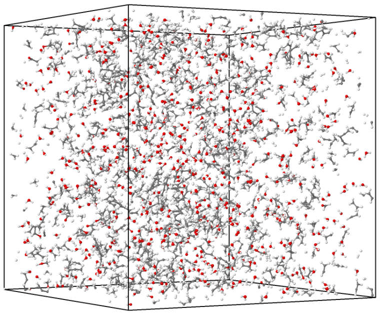

(d) t = 300 ps

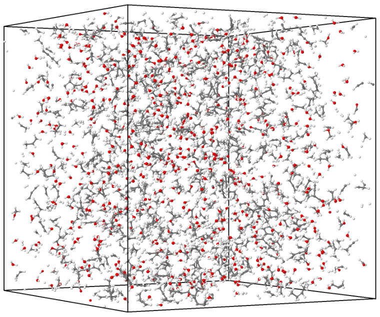

(e) t = 400 ps

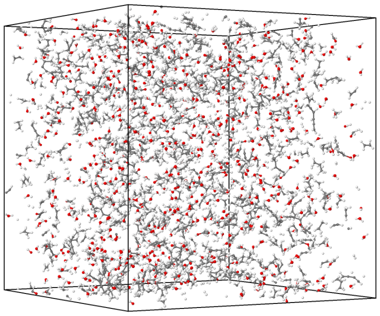

(f) t = 500 ps

Figure S4: Selected snapshots of the HTG process of PE (system 4) at T = 2500K.

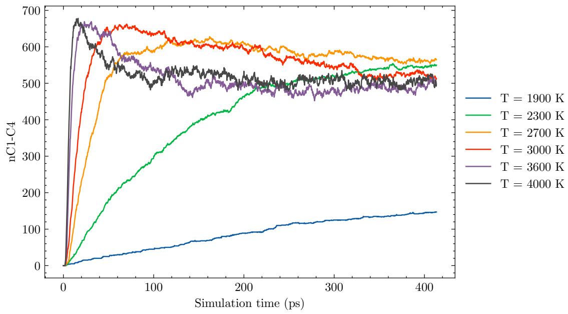

(a)

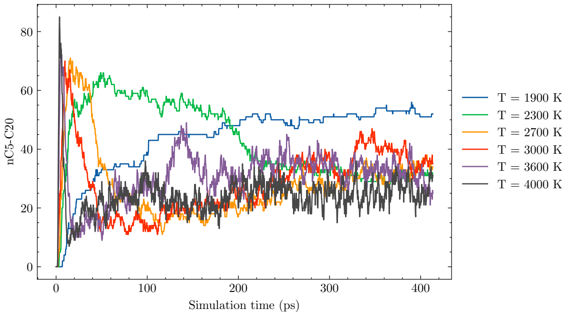

(b)

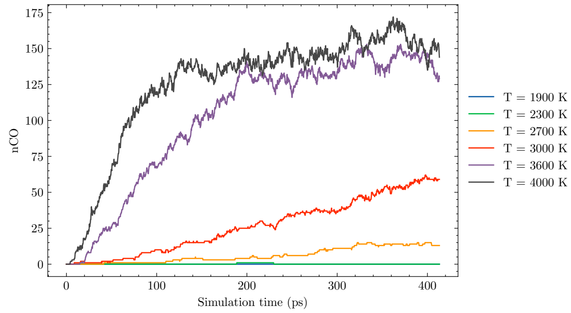

(c)

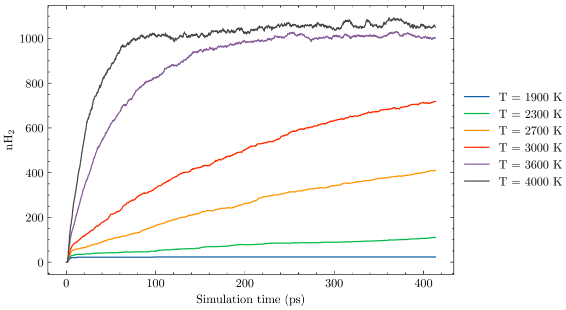

(d)

Figure S5: Selected snapshots of the HTG process of PE (system 2) at T = 2500K.

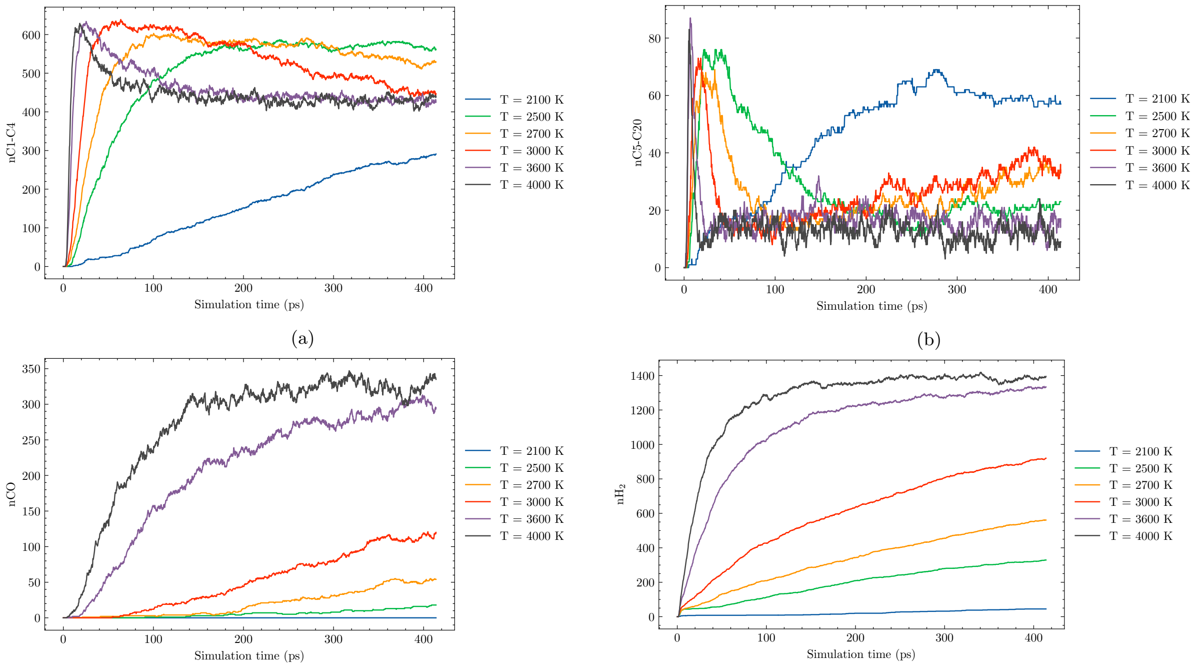

(a) (b)

(c) (d)

Figure S6: Selected snapshots of the HTG process of PE (system 4) at T = 2500K.

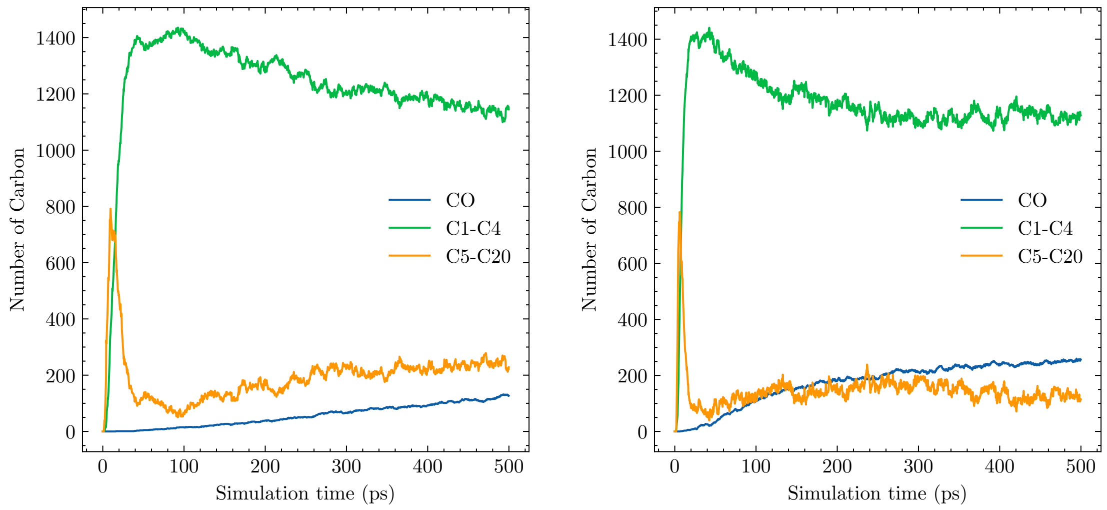

(a) (b)

Figure S7: Number of carbon atoms in different product during HTG process at temperature T = 3000 K (a) and T = 3600 K. At these temperatures, the C1-C4 product is partially converted into CO and C5-C20.

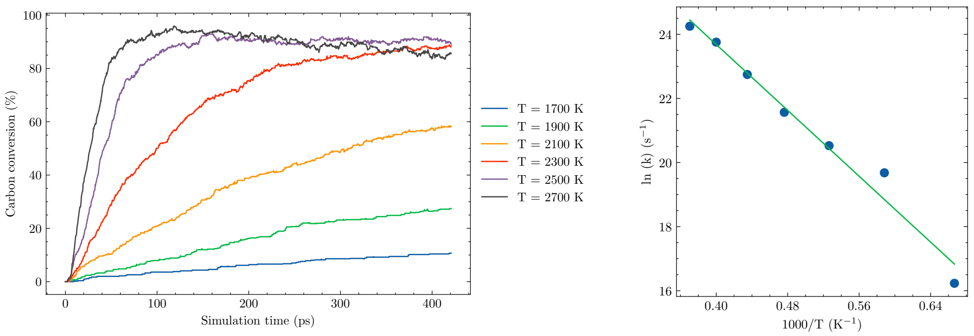

(a) (b)

Figure S8: Carbon conversion of system 2 during HTG process at different temperatures (a) and the plot of logarithm of rate constant as a function of the inverse temperatures (b).

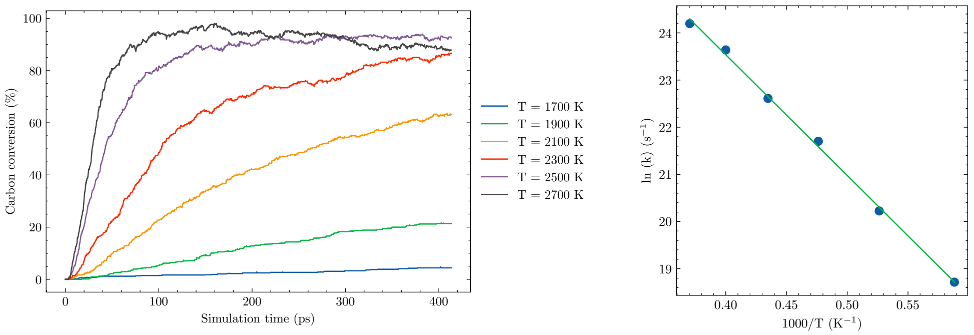

(a) (b)

Figure S9: Carbon conversion of system 3 during HTG process at different temperatures (a) and the plot of logarithm of rate constant as a function of the inverse temperatures (b).

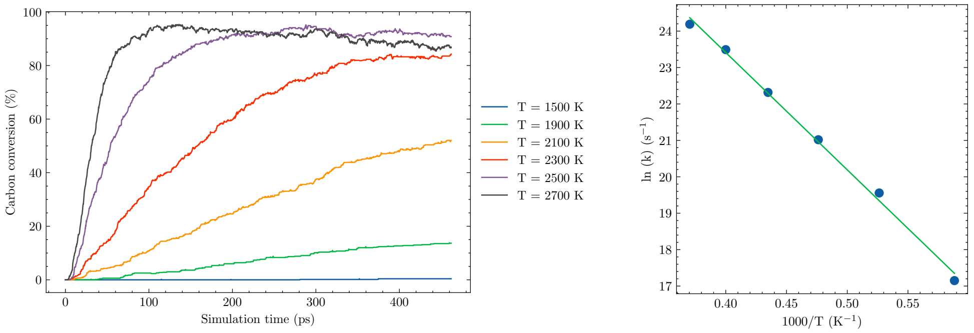

(a) (b)

Figure S10: Carbon conversion of system 4 during HTG process at different temperatures (a) and the plot of logarithm of rate constant as a function of the inverse temperatures (b).
